# Spanning the boundaries between policy, politics and science to solve wicked problems: policy pilots, deliberation fora and policy labs

**DOI:** 10.1007/s11625-022-01187-y

**Published:** 2022-08-23

**Authors:** Ulrike Zeigermann, Stefanie Ettelt

**Affiliations:** 1 Institute of Political Science and Sociology, Julius-Maximilians-Universität of Würzburg, Wittelsbacherplatz 1, 97074 Würzburg, Germany; 2 Department of Health Services Research and Policy, London School of Hygiene and Tropical Medicine, 15-17 Tavistock Place, London, WC1H 9SH UK

**Keywords:** Boundary spanning, Policy pilot, Deliberation, Policy lab, Health policy, Climate policy

## Abstract

Current crises have highlighted the importance of integrating research, politics and practice to work on solutions for complex social problems. In recent years, *policy deliberation fora,  policy pilots* and *policy labs have increasingly been deployed* to mobilise science to produce solutions, help create popular support and guide implementation of policies addressing major public policy problems. Yet, we know little about how these approaches manage to transcend the boundaries between research, politics and practice. By systematically comparing policy deliberation fora, policy pilots and policy labs, this paper explores their mechanisms of boundary spanning including relationship and trust building, knowledge translation and developing solutions. We situate our analysis in healthcare policy and climate change policy in Germany, two contrasting policy fields that share a perpetual and escalating sense of crisis. Our findings suggest that deliberation fora, policy pilots and policy labs address different dilemmas of policymaking, namely the idea dilemma, the implementation dilemma and the legitimacy dilemma. All three approaches reduce wicked problems to a manageable scale, by grounding them in local decision-making, reducing their scope or reducing the problem analytically. We argue that despite their ambition to modernise democratic practices, unless they are institutionally well embedded, their effects are likely to be small scale, local and temporary.

**Supplementary Information:**

The online version contains supplementary material available at 10.1007/s11625-022-01187-y.

## Introduction

Wicked problems result from a conjunction of global and interrelated causes, have dynamic and often unforeseen consequences and involve a large set of actors (Head and Alford 2015; Peters 2017; Rittel and Webber 1973). Recent responses to wicked problems have seen a variety of novel approaches to mobilising science. These include approaches as diverse as policy deliberation fora, policy pilots and policy labs that are the focus of this study. We understand these approaches to problem solving as recent boundary spanning activities aimed at transcending boundaries between science, politics and practice. To date, it remains unclear how they contribute to solving wicked problems and how they foster the exchange between science, politics and practice. Discussing examples of all three approaches, the paper explores how they span these boundaries and how they aim to contribute to solving wicked problems.

In Germany, policy deliberation fora, policy pilots and policy labs are increasingly deployed, driven by a growing sense of urgency to address long-term problems of ensuring the sustainability of publicly funded health services and climate mitigation. By systematically comparing three types of boundary spanning activities used for tackling major public policy problems, this paper seeks to contribute to current debates on the role of science in politics and practice in a time of multiple crises (Böcher and Zeigermann 2021; Hulme et al. 2020). It uses two pertinent policy fields in Germany—health-care delivery and climate change—to investigate how deliberation fora, policy pilots and policy labs are deployed to tackle wicked problems and to address the dilemmas associated with such problems. Germany provides an interesting country context due to the amount of policy activity in both policy fields, a willingness to engage in these relatively novel approaches to policymaking (although the novelty may be greater in Germany than in other countries) and an awareness of the limitations of traditional policymaking. We show that these activities address key dilemmas policymakers face when dealing with wicked problems, i.e. by shrinking problems to a manageable scale, by ‘localising’ them, reducing their scope or reducing the problem analytically.

In the following section, we discuss the concept of boundary spanning and how it can be utilised to analyse the role of activities for addressing wicked problems. This is followed by a description of our methods, the presentation of our findings and a discussion of key insights from this study. We aim to show how boundary spanning activities transcend subsystems to generate novel ideas, reduce implementation risks and increase popular support, which we conceptualise as the three main dilemmas of wicked problems.

## The mechanisms of boundary spanning activities

With many countries facing increasing pressures from urgent climate change and public health problems, there has been a burgeoning interest in the role of boundary spanning in solving wicked problems. In the policy sciences, the concept of boundary spanning has been used in the analysis of how organisations can collaborate productively across different policy fields and subsystems to achieve common goals (Sheikh et al. 2016; Jochim and May 2010; Sabatier 1991) and how scientific knowledge can be leveraged to improve policy decisions (Bednarek et al. 2018). Although the concept is by now well established, it has also been criticised as being vague and difficult to operationalise. There is no agreed definition of the nature of subsystems and it is not always clear what constitutes the boundary between them (Faling et al. 2016). The concept has also been criticised for blurring the boundaries between different forms of knowledge and thereby discrediting or politicising independent scientific evidence and for neglecting critical power relations in interactions between actors from different subsystems (Böcher and Krott 2016; Pielke 2004; Ruppert-Winkel et al. 2015).

Policy scholars have used the concept of boundary spanning to analyse efforts to develop policy aimed at wicked problems. These studies propose, for example, to address wicked problems through new forms of leadership and stakeholder involvement that elicit collaborative solutions by integrating multiple perspectives (Head and Alford 2015; Williams 2013) or through new forms of public organisation that promote creativity and flexibility (Decastri and Buonocore 2021). They also examine the role of science and the challenges of working across the science, policy and practice interface to develop solutions to complex policy problems (Bednarek et al. 2018; Horton and Brown 2018; Jasanoff 2016; Lemos et al. 2019; Posner and Cvitanovic 2019). Boundary spanning hereby entails diverse activities that can be structured along three main types of activities that are dependent on their institutional setting as well as stages in the policy process.

### Boundary spanning as relationship and trust building

Building relationships and trust are at the heart of activities that span the boundaries between organisations, subsystems and policy fields (Posner and Cvitanovic 2019, 144). Studies have shown that actors who know each other are more likely to trust one another (Chen et al. 2014; Coleman and Stern 2018; Kucharska 2017; Song et al. 2019). In a similar vein, people are more likely to accept information if they trust the messenger, even if the evidence contradicts their initial assumptions. This way, trust becomes a crucial ingredient in bringing actors together to collaborate across boundaries to solve complex problems. One strategy to hold networks together is to create joint projects, which give them purpose, focus their energies and help build trust (Brouwer and Biermann 2011).

Relationship building also means deliberately connecting diverse forms of knowledge and working towards an alignment of values, goals and purposes associated with different subsystems (e.g. science, practice and politics). This increases the legitimacy of knowledge for dealing with wicked problems, but may also come at a cost to individual network members who expose themselves to criticism and confrontation (Cash et al. 2003; Gibbons 1999; Nowotny 2003). Intentionally or unintentionally, networks can exclude certain forms of knowledge or minority positions in research, practice or politics as a consequence of the process of selecting network members (Zeigermann 2021). It is therefore also important to understand who has access to these networks, whose positions may be under-represented and who is entirely left out.

### Boundary spanning as knowledge translation

Boundary spanning activities typically involve different forms of knowledge translation (Hassenteufel and Zeigermann 2021; Leith and Vanclay 2015; Wright and Nyberg 2016). At a basic level, this means ensuring effective communication, information flows, and conveying complex ideas, concepts and framings of problems that are established among one group of actors but are unfamiliar to actors from different institutional and ideational backgrounds (Aldrich and Herker 1977; Tushman and Scanlan 1981; Williams 2013). Boundary spanners help actors on both sides of the boundary obtain information that would not be accessible otherwise, to “speak the same language” and therefore reduce miscommunication (Tushman 1977).

These insights emphasise the transactional aspect of knowledge translation. Studies have shown that knowledge translation is more successful if boundary spanners are able to link new knowledge to previous experiences and existing knowledge of policymakers (Failing et al. 2016). Other techniques include developing narratives, telling stories or shifting the narrative frame to make information more relevant and relatable (Stone 2019). This process has also been described as a transformation of ‘capitals’ (Bordogna 2019; Kislov et al. 2017), a concept introduced by Bourdieu’s theory of social fields (Bourdieu 2006). Capitals can be understood as the resources that actors possess and pursue within a subsystem. Scientific evidence is one of these resources and it can strengthen the authority and legitimacy of a policy proposal. However, this requires that the source of the evidence is trusted and seen as legitimate. As a boundary activity, efforts to increase the impact of research on policy not only need to communicate research findings to policymakers, but also enable policymakers to access, engage with and see the relevance of research. However, studies have shown that some types of research are easier to communicate than others and have more resources for boundary spanning activities, leading to findings from some disciplines and in some policy areas being more often and more effectively translated than in other disciplines and areas (Bartl 2022; Victor 2015; Zeigermann 2022). The translation process can therefore not be seen as neutral, but as a strategic activity of interpreting and producing information (Hassenteufel and Zeigermann 2021) that may have an impact on the understanding of, and development of solutions to, wicked problems.

### Boundary spanning as developing solutions

Finally, most boundary spanning activities in policy networks aim at the development of novel policy solutions (Zeigermann 2021). Creative processes contribute to exploring new policy solutions, constructing new ideas and “breaking out of habitual and common problem definitions” (Tippmann et al. 2017, 456). For example, Nederhand and colleagues highlight the innovative component of boundary spanning activities for “changing existing routines” or “creating turns” by attempting to recognise and exploit policy windows (Nederhand et al. 2019, 224).

Policy solutions can be the product of networking activities and knowledge translation (Arnott et al. 2020; Leith and Vanclay 2015; Wright and Nyberg 2016). However, effective boundary spanning requires the integration of knowledge at the right time in the policy process (Böcher and Krott 2016, 44; Posner and Cvitanovic 2019, 143; Stjerne et al. 2019). This means that the potential of novel solutions depends on the support of policy actors and the wider context of policymaking (Bednarek et al. 2018). Transcending the boundaries between science, policy and practice is thus inherently political, as it challenges or reinforces the existing distribution of power (Kislov et al. 2017). Thus, there are important challenges and potential biases to consider when analysing the solutions developed for wicked problems through boundary spanning.

To conclude, the previous paragraphs outlined how policy scholars operationalise boundary spanning activities. They can be clustered into three main types, including relationship and trust building, knowledge translation and developing policy solutions.

## Research design and case selection

**Fig. 1:**
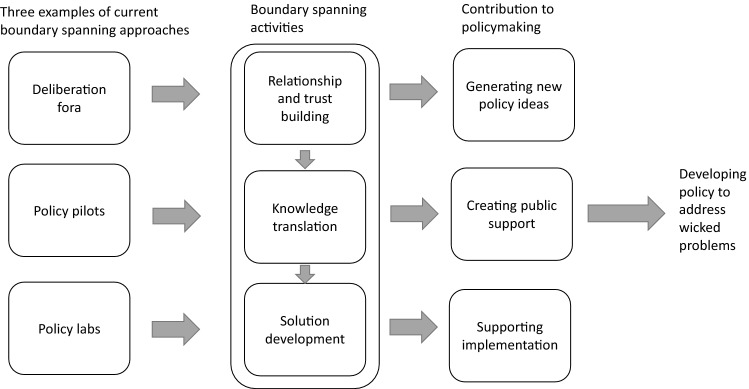
Analytical framework for examining boundary spanning activities to solve wicked problems

In this paper, we examine how boundary spanning activities are deployed in novel policy approaches to address wicked problems. We focus on deliberation fora, policy pilots and policy labs as typical cases for such novel approaches. They seek to mobilise science to tackle wicked problems at different stages in policymaking, although they are not necessarily sequentially connected and will therefore be studied individually. Drawing on public policy analysis (e.g. Howlett et al. 2009), we consider boundary spanning activities as differing across the diverse stages in the policy process. First, policy deliberation fora bring together a group of people, randomly selected and intended to be representative of a population, to enable mutual learning and generate policy recommendations based on information provided by experts. Their main aim is to enhance citizen engagement with policy problems and to develop “alternative arguments with an open mind” (Niemeyer 2013, 435) in a context of increasing dissatisfaction with representative democracy (Flinders and Curry 2008), political polarisation and the difficulties of political engagement in the age of social media (Dryzek et al. 2019). They engage citizens in informed agenda setting and policy formulation (Duvic-Paoli 2022). Second, policy pilots are time limited and implement policy at a small scale in a limited number of places (Ettelt and Mays 2019). They sit between policy formulation and policy implementation and allow for small-scale testing and learning by ‘trying out’ to inform future policy decisions. Third, policy labs have emerged in recent years as new organisational entities that aim to bring innovation to public policy (Asenbaum and Hanusch 2021). There is no clear definition of what policy labs are or what they do (Olejniczak et al. 2020). Broadly speaking, the term ‘policy lab’ signifies a space intended for the production of novel policy ideas, usually through collaborative and participatory methods. The following Fig. 1 summarises our analytical framework.

We consider boundary spanning activities as varying across different policy domains with different institutional structures, ideas, actors, forms of interactions and connections with knowledge for each subsystem of public policy (Howlett et al. 2009). Accordingly, we analyse how policy pilots, deliberative fora and policy labs are deployed to address wicked problems in the two pertinent fields of health and climate policy. Both policy fields have significantly changed over the last decade in a context of climate change, demography and the COVID-19 pandemic. Due to persisting contestation of the problem definition and problem solution as well as high levels of complexity and uncertainty, both health and climate problems are typical wicked problems (Lawrence 2020; Levin et al. 2012).

Deliberation fora, policy pilots and policy labs have been introduced in climate and health policy in many European countries. For our empirical analysis of boundary spanning activities, we focus on processes in Germany since 2015 because it provides an interesting context for this analysis. Due to its federalist constitution, solutions to policy problems in Germany typically require collaboration of several levels of government including federal, state and often district authorities (Fleischer and Carstens 2021, 2; Head and Alford 2015, 716). There are currently few analyses of boundary spanning activities in health and climate change policy in Germany despite the fact that both are highly dynamic policy fields characterised by a large number of policy actors, substantial pressures from organised interests, enormous economic and social complexity, and increasing scepticism of citizens as to whether traditional approaches to policymaking are effective in producing solutions.

As deliberation fora, policy pilots and policy labs were established only relatively recently, there exist only a few reports from government about these approaches. We therefore used media reports and the research literature, in addition to our own knowledge of the policy areas, to identify relevant empirical cases of boundary spanning activities in Germany, by systematically searching the Factiva and Scopus databases using the search terms ‘policy lab’, ‘policy pilot’, ‘deliberative forum’ (and synonymous terms such as ‘townhall meeting’, ‘mini-publics’, ‘citizen assembly’) and ‘climate’ or ‘health’ in English and German. Both researchers were involved in this initial search process from March to April 2021. Results from the individual preliminary search were then compared and adjusted until a first selection of relevant articles was agreed. We identified 205 relevant media reports and eight research articles (see Online Appendix 1 describing the search flow diagram). In the following 2 months, cases mentioned in documents were manually screened and coded according to their policy field, issue focus, level of implementation, source of funding, actors initiating the activity, implementation period and link to science (see Online Appendix 2 providing an overview on the cases). For this purpose, we also used additional information about these cases on websites, in government reports or press statements from organisations to gain a comprehensive overview. Boundary spanning approaches beginning before 2015 were excluded.

Data collection and analysis has proven to be challenging due to the proliferation of activities and the labels used to signify novel approaches. ‘Policy labs’, in particular, include a variety of approaches, ranging from the use of particular approaches such as design thinking, to more traditional workshop or meeting formats, and in some instances including or being synonymous with pilots (e.g. digitalisation labs). Pilots and fora also are organised in many different ways, complicating the delineation of cases. Cases were limited to those initiated at federal level, to allow for an analysis of the multi-level nature of these approaches.

## Analysis of boundary spanning activities

In what follows, we analyse the boundary spanning activities of policy deliberation fora, policy pilots and policy labs. We illustrate our findings presenting a single case per approach, selected for their prominence in the field, and their relevance to this analysis, as well as their multi-level nature and complexity. We analyse these cases by examining how these approaches span boundaries between politics, practice and science by facilitating relationship and trust building, supporting knowledge translation and developing policy solutions.

### Policy deliberation fora

The “Bürgerrat Klima” (Climate Citizen’s Assembly) serves to illustrate the use of policy deliberation fora. Our review of the literature and media has shown that it is only the third nationwide deliberation forum and the first climate citizen’s assembly in Germany. To our knowledge, no deliberation forum exists in health policy organised at the national level. However, as of June 2021, two fora were organised on health policy topics at the regional and local level, covering topics like vaccination and post-pandemic health care. In addition, 13 deliberation fora were organised on climate-related topics, including climate mitigation and adaptation or energy transition at the regional (*n* = 7) and local level (*n* = 6).

Starting in April 2021, the German Climate Citizen’s Assembly has been inspired by previous policy deliberation fora in climate policy in Ireland, France and in the UK (Duvic-Paoli 2022; Willis et al. 2022). It emerged out of the lively public debate on the German Climate Mitigation Act (Klimaschutzgesetz 2019/2021) that was significantly influenced by climate movements such as ‘Fridays for Future’. ‘Fridays for Future’, together with ‘Scientists for Future’ and other civil society groups, increased political pressure on policymakers to address climate change and demanded greater participation of citizens, including young people and other groups in society who are normally little involved in policymaking (S4F 2020). The German Climate Citizen’s Assembly was set up by the civil society organisation “BürgerBegehren Klimaschutz” and implemented independently through three private agencies, supported by an expert advisory board of 25 senior researchers and a civil society advisory board. The Citizens' Assembly gathered 160 randomly selected citizens (contacted over telephone, representative in their composition of age, gender, education, geographic background, migration background characteristics). Twelve meetings were held over the summer in 2021 to discuss four areas of action (transportation, buildings, energy production and food), determined by the advisory boards. At the end of the meetings, participants voted on policy recommendations to be submitted to the political parties in the German Bundestag. The final report was published before the federal elections in September 2021 so that the recommendations could be considered in the coalition negotiations.

#### Relationship and trust building

The German Climate Citizen’s Assembly was first and foremost aimed at bringing together diverse actors affecting (and affected by) climate policy who would usually not come together to discuss complex political problems and their solutions. The deliberation forum thus mainly intended to play a key role in contributing to building trustful collaborations by emphasising the role of informed decisions. By involving people with diverse backgrounds and interests that are directly affected by climate policy solutions, the idea was to promote popular support for contested problem definitions and decisions on climate mitigation and adaptation. This required, however, extensive, time-consuming and resource-intense preparation to facilitate the debate among diverse participants and communicate the outputs of the Citizen’s Assembly to policymakers and the public to produce effects. Heads of the main political parties received the recommendations developed by the Citizen’s Assembly in summer 2021. As a consequence, the 2021–2025 Coalition Agreement supports deliberation fora as an instrument for a “living democracy” and an additional form of dialogue with citizens in a representative democracy (SPD, Bündnis 90/Die Grünen, FDP 2021, 8). At the same time, participation in such deliberation fora remains limited to a small group of randomly selected persons who cooperate and network for building trustful relationships. Furthermore, due to the organisation of deliberation fora, the understanding of the complex interdependencies related to a wicked problem like climate change is reduced by the necessary division of the whole group into smaller thematic groups, so-called “action areas”.

#### Knowledge translation

With the support of learning processes across stakeholders from politics, practice and science, the forum sought to contribute to translating diverse experiences and forms of knowledge. The underlying idea of the German Climate Citizen’s Assembly was that initial problem definitions of stakeholders are transformed and options for solutions linked to scientific research and prognoses. Scientific experts were expected to facilitate translation processes across practice and politics with the help of research. The composition of the group of experts shows that diverse scientific disciplines were gathered to provide information from different perspectives (Bürgerrat Klima 2022). A central purpose of the selection process of participants was to overcome—or at least address—major conventional selection biases and power relations in policy processes. While a filtering of information certainly also takes place in deliberation fora, trust-building efforts seek to contribute to broader acceptance, and the specific processes to greater transparency about institutional, conceptual and political translations of knowledge. Experiences with the German Citizen’s Assembly indicate that this deliberation forum was only partly successful. A public survey initiated by the BürgerBegehren Klimaschutz e.V. and undertaken in June 2021 found that almost 80% of the respondents agreed that the recommendations from the German Citizen’s Assembly should be taken up by the German government (Forsa 2021) and another study confirmed that citizens broadly supported the outcome of the Citizen’s Assembly (Betsch and Sprengholz 2021). At the same time, the survey also found that about 75% of the respondents had never heard about the German Citizen’s Assembly on Climate (Forsa 2021) and it remains unclear to what extent people who criticize climate science were included in the German Citizen’s Assembly.

#### Developing solutions

The rationale behind the German Climate Citizen’s Assembly was that the deliberation forum also produces innovative solutions by addressing issues of legitimacy in climate policy processes. For that purpose, smaller groups of 40 participants discussed issues such as transportation, buildings, energy production or food to develop accepted and novel solutions for each action area (Bürgerrat Klima 2021). Integrating diverse perspectives brings about new policy recommendations for recognised complex problems and can re-evaluate underlying paradigms determining conventional solutions. Fora, therefore, can produce alternatives in a context of increasing dissatisfaction with representative democracy. At the same time, most recommendations from the Climate Citizen’s Assembly do not address the (potentially conflicting) interactions. They do not provide a prioritisation, but present recommendations as a holistic set of policy measures for each action area. Complex climate problems were reduced to a manageable scale in action areas, yet, participants were partly overwhelmed with the complexity of information shared with them and the time frame and setting did not allow them to develop real alternatives to the siloed approach generally found and criticised in regard to wicked problems. The overall guidelines (Leitsätze) of the Climate Citizen’s Assembly remain therefore very general. They reflect the vision of the 2015 Paris Agreement and the 2021 German Climate Mitigation Act.

To conclude, the case of the “Bürgerrat Klima” illustrates that policy deliberation fora bring together diverse people so that practical experiences of citizens can contribute to developing legitimate policy solutions to wicked problems. Solution development is supported by expertise that provides prognoses, research on causes and interdependences, and learning tools related to the topic being discussed. At the same time, the German Citizen’s Assembly also shows that deliberation fora remain today minor (resource-intense) initiatives for increased public dialogue and knowledge translation that have so far only limited visibility and—if any—indirect influence on policy-making and implementation.

### Policy piloting

In Germany, there is a proliferation of policy pilots at regional and local levels in both climate and health policy (including pilots of pandemic-related policies). A prominent example of piloting initiated at the national level is the ‘Innovationsfonds’ (Innovation Fund) in health care. The Fund was set up in 2015, following the introduction of the Act to Strengthen the Supply of Health Care (GKV-Versorgungsstärkungsgesetz). The act mandates the self-administration in health care, constituted by the national associations of sickness funds, hospitals and office-based doctors, to dedicate funding to pilot projects that test novel approaches to healthcare provision, as well as to health services research. The aim of the fund is to stimulate ideas for novel approaches and test these in practice at a small scale over an agreed period of time. Projects could be suggested by various actors including provider organisations and sickness funds following a number of themed or open calls for proposals from the Fund. Evaluation is mandatory for all pilot projects (Blettner et al. 2018). In December 2020, over 150 pilot projects had been commissioned aimed at testing novel approaches to organising health services, in addition to over 260 health services research studies (Deutscher Bundestag 2020).

#### Relationship and trust building

The Innovation Fund has an explicit role in supporting collaborations between the actors in healthcare, especially those involved in healthcare provision (e.g. hospitals, ambulatory practices) and sickness funds, as well as others not usually represented in health policymaking (e.g. patients, providers of preventative and social services). Actors participating in local projects align their goals and purposes when testing ideas through local implementation, even though as payers and providers they are often not in agreement on policy questions. There are two dimensions of relationship building in piloting through the mechanism of the Innovation Fund. At the horizontal level, actors cooperate within their local networks and share knowledge and experiences, both informally and formally (e.g. through project monitoring and evaluation). Through vertical boundary spanning, Innovation Fund projects provide an opportunity for direct knowledge exchange between implementers and policymakers of the self-administration through evaluation and project reporting that feeds into policy decisions (e.g. transfer into routine practice).

#### Knowledge translation

Projects funded through the Innovation Fund are specifically aimed at testing novel ideas and translating them into local practice. It is a condition of project funding that each pilot is scientifically evaluated and that the evaluation is conducted separately from the implementation of the measures that are piloted. Evaluation is, in principle, a key instrument of translating knowledge across the boundaries of practice, policy and science: to translate ideas of diverse, including international, origin into local practice, local practice into experiential knowledge and evaluation findings, and potentially into national-level policy decisions, such as the inclusion into the national service catalogue. In Germany, there is now a sizable number of organisations offering evaluation as well as an established multi-disciplinary tribe of academic researchers involved in delivering health service evaluations which the Innovation Fund helped to develop. In the case of the Innovation Fund, there are several avenues for knowledge translation through evaluation. The Innovation Fund is aimed directly at informing policy decisions taken by the Federal Joint Committee (Gemeinsamer Bundesausschuss), the highest decision-making body in the German self-administration in health care, and other health policy decision-makers. Indeed, the Innovation Committee is required to assess each project and evaluation report within a given period of time and make a recommendation for or against policy change (e.g. by including the new form of service delivery into the reimbursement schedule or adjusting regulation). A second avenue for knowledge translation is to encourage learning from the experience of conducting the pilots, and their evaluation, through the publication of project and evaluation reports. In addition, many research teams have published their findings in peer-reviewed journals (Heytens et al. 2021). A third avenue of knowledge transfer is learning from being evaluated by the teams that conducted the pilots, although this form of translation is highly dependent on whether the measures piloted will be continued in the future, which often requires an enabling decision at policy level.

#### Developing solutions

Pilot projects are ostensibly aimed at developing policy solutions. Their contribution to finding solutions lies in their approach to practical testing. The Innovation Fund provides consortia with the opportunity to develop implementation strategies and adopt ideas to their local context. However, its main aim is to draw lessons from these experiences and generate evidence in support of national expansion. The Innovation Fund therefore provides an example of two types of policy solution through piloting: at local level, it allows for selectively supporting individual projects to test novel ideas in practice, with the aim of understanding their implementation. At the national level, the Innovation Fund provides a mechanism to overcome the perceived lack of innovation in the German healthcare system and to generate knowledge to implement policy and practice change. While it is outside the remit of the Fund to support implementation beyond the period of piloting, its stated aim is to support the development of solutions to improve healthcare provision (Gemeinsamer Bundesausschuss 2022).

In sum, the case of the Innovation Fund illustrates that there are two boundaries being transcended in policy piloting. The first boundary is transcended between policy and practice and allows for learning from experience through pilot implementation. The second boundary is spanned in a reverse movement from practice to policy, which is facilitated through the evidence produced in the process of piloting. This more formal learning assesses the outcomes of the pilots, i.e. the effects of the interventions that have been piloted, the processes involved in their implementation or a combination of both (Hayes et al. 2014; Jowell 2003). This means that the scientific contribution to piloting is embedded in the process of pilot implementation. However, these two forms of learning can produce friction, e.g. when local implementers are convinced of the benefits they experienced, but the measurable outcomes are insufficient to support a decision to continue (Ettelt et al. 2015).

### Policy labs

In Germany, policy labs are mainly set up at the local level to develop sustainable solutions for transitions in energy generation or transport, for instance, through technical and digital applications. Broadly speaking, the term ‘policy lab’ signifies a space intended for the production of novel policy ideas, usually through collaborative and participatory approaches (Asenbaum and Hanusch 2021). Some ‘labs’ are based in existing organisations, but the term is also used for projects, programmes or events. Many policy labs operate within a specific policy field (e.g. biodiversity, climate change, technology), while others take a broader view on public policy.

The 2017 Online Access Act (OZG), which requires the administration at the federal, regional and district level to offer digital administrative services by the end of 2022, established for the first time nationwide policy labs for administrative agencies (BMI 2021). In total, 52 digitalisation labs were set up for a period of 3–6 months. They are generally organised by non-governmental organisations and financed by the Federal Ministry of the Interior, Building and Community (Mergel 2019). Their main objective is to develop user-friendly digital access to public services (BMI 2019, 41; Fleischer and Carstens 2021, 6). For that purpose, several strategies are used in the labs, including bringing together policy actors from all administrative levels, while at the same time limiting the number of actors involved and establishing flat hierarchies (Carstens 2021, 12). The solutions designed in these labs will then be made available to all federal states to allow for harmonisation between state administrations (ibid., 95).

#### Relationship and trust building

Policy labs are specifically aimed at bringing together people across the spectrum of actors involved in (or exposed to) policy. They are typically associated with participatory methods to include the views of citizens and local communities into policy design (Sørensen and Torfing 2015). Some digitalisation labs consist of ten participants, including civil servants, digitalisation experts, product owners, legal experts, designers and service users, in design-thinking and Scrum-oriented formats (BMI 2019, 28; Carstens 2021, 10). These policy labs are therefore highly exclusive. This stands in contrast to the rhetoric of participatory stakeholder engagement, since only a small number of purposely selected persons take part in these labs. Meeting facilitators structure the exchange of information and a solution-development process and encourage participants to contribute their skills (Fleischer and Carstens 2021). They also have the power to decide who has agency in those labs and who does not. This process can amplify power asymmetries across different stakeholders, because generally only those persons who are already part of a network are invited. Their contribution to the debate is then—through the facilitation of the exchange and focus on innovation design—to an extent dependent on their expected suitability and contribution to achieving a desired output. Thus, trust is a precondition of the process, as well as its outcome. Relationship building therefore takes place mostly among a small but powerful group, while the different disciplinary background and knowledge of the participants serve to promote popular support to the solutions developed in innovation labs.

#### Knowledge translation

Policy labs tend to value creativity over technical expertise and imagination over evidence, although this will be difficult to generalise given the diversity of approaches (Lewis et al. 2019). Design thinking comes with a set of collaborative techniques that encourage and enable participants to develop creative solutions to policy problems. This involves reducing the problem to key principles to shift the policy perceptions, to help move to a more manageable and open problem-solving strategy and avoid getting bogged down in the usual quagmire of complexity and detail associated with implementation (Dorst 2011). In the digitalisation labs, digital solutions to service provisions were developed by bringing together different types of administrative, technical and legal knowledge as well as user experience. Although policy labs using design thinking approaches do not purport to be informed by science and research, knowledge translation occurs in other ways by spanning boundaries between different types of knowledge and experiences provided by participants.

#### Developing solutions

The aim of policy labs is to generate new ideas and develop creative solutions to policy problems through bringing in different forms of expertise and experience. In contrast to policy pilots, they do so by emphatically reducing the complexity of problems to make them manageable for a designed solution. They specifically aim to shift the perception and framings of problems to create space for novel solutions. Their main contribution therefore lies in generating new ideas that can be used, or at least tested, in practice to solve policy problems. In our example, digitalisation labs focussed on specific services (e.g. developing a mobile app) rather than attempting to solve the problem of improving digitalisation of public services in general, reducing the problem to be solved to a specific set of administrative, technical and legal questions. Other labs may engage in ‘prototyping’, but engaging with the intricacies of complex implementation processes is usually not their core business.

In the literature, a unifying theme of policy labs is that they aim to solve “the social and public problems that vex governments” (Williamson 2015, 4)—thus they are firmly in the business of contributing solutions to wicked problems. The laboratory metaphor implies that policy labs are organised around a set of specific methods to achieve their aims. Both in climate and in health policy, collaborative approaches are aimed at organising the interactions between participants to facilitate the joint production of novel ideas and policy solutions. Policy labs in Germany are often discussed in combination with design thinking as a novel approach to generating innovative ideas, although in the example of ‘digitalisation labs’ this is not obvious. The facilitators of policy labs have a powerful role in the process and in moderating the contributions from participants, including those representing science. In that regard, we have critically discussed the exclusive character of policy labs, but also noted that policy labs are inclusive of other positions than in traditional policymaking. It remains unclear to what extent transcending the boundaries between different forms of knowledge and experience provided by the selected participants can ensure transferable and long-term outcomes, considering that the effective implementation of novel approaches heavily depends on the political and organisational setting.

## Discussion and conclusion

The analysis shows that deliberation fora, policy pilots and policy labs in climate and health policy engage in all three boundary spanning activities to address wicked problems. First, they engage in relationship and trust building by bringing together different groups of actors. Deliberation fora are most obviously invested in increasing diversity of participation using the mechanism of randomly selecting participants. Labs also emphasise the diversity of perspectives, yet this tends to be reflective of the expertise and perspectives that individual members of a lab bring to the table. In both cases, the group of participants are relatively small, enabling a degree of trust building and alignment of purpose, although it is unclear whether this exceeds the duration of the meetings. During policy pilots, relationship building may be more lasting, usually over the duration of months or years, but pilot projects tend to collapse when the funding runs out, unless more durable arrangements can be found.

Second, labs, pilots and fora involve strategies for knowledge translation, typically including scientific knowledge. Deliberation fora related to climate policy are heavily informed by science to set out the causes of climate change and frame possible routes to action. In contrast, policy piloting, through evaluation, is a vehicle for generating empirical scientific knowledge, again with emphasis on relevance for policy and practice. The pilots themselves may be aimed at producing scientific evidence (as in the Innovation Fund), but not every pilot does so. It is less clear how policy labs draw on scientific research and practices, as the focus of this activity is on finding novel solutions through creative design processes, deemphasising the role of research potentially. Crucially, they also draw on other sources of knowledge including lived experience (e.g. of patients and citizens) and practical knowledge (e.g. of implementers and professionals). In our example, policy labs and deliberation fora made specific attempts to involve groups that are not usually participants in policy processes, for example by randomly inviting citizens to participate in townhall meetings. The question is then whether any of these activities change the balance of power associated with different types of knowledge. While deliberation fora and labs certainly set out to level the playing field, their success will depend on how they are conducted in practice, how they are organised and facilitated, and ultimately whether they have any influence on actual policy decisions.

Third, the three approaches contribute differently to the development of policy solutions. Deliberation fora seek to develop solutions by involving local populations in policy development and by doing so to increase the legitimacy of policy decisions. Policy pilots aim to develop solutions by trying out and testing existing ideas for service improvement, with the aim of focussing policy decisions on the most successful candidates and strengthening their implementation. Policy labs aim to generate novel ideas to solve problems by tapping into the creativity and diverse experience of participants.

Although there are limitations to desk research as interviews could provide more detailed information about the mechanisms of boundary spanning, their (strategic) use and the experiences within a specific project, our analysis suggests that through their boundary spanning activities, deliberation fora, policy pilots and policy labs are equally important as they address different dilemmas of policymaking. They transcend the boundaries between science, politics and policy at different stages in the policy process, setting different priorities and using different mechanisms for networking, knowledge translation and solution development. We could not identify significant differences in boundary spanning activities between the two policy domains of health and climate. As summarised in Table 1, all three approaches aim to generate novel policy ideas (addressing the ideas dilemma), improve their implementation (implementation dilemma) and increase the legitimacy of, and thus public support for, policy change (legitimacy dilemma).

**Table 1:** How boundary spanning activities address wicked problem dilemmas

Wicked problem dilemma/boundary-spanning activity	The ideas dilemma	The legitimacy dilemma	The implementation dilemma
Relationship building	Involve actors that are usually excluded from the policy process (policy labs)	Include citizens directly affected by policy solutions (deliberation fora)	Explore new forms of collaborations across organisations and sectors levels (policy pilots)
Knowledge translation	Inclusive of different types of knowledge, including scientific knowledge, to generate ideas (policy labs)	Evaluation helps generate scientific knowledge to inform and legitimise novel approach and risk taking (policy pilots)	Learning from diverse experiences and knowledge of stakeholders contributes to broader acceptance that should reduce implementation problems (deliberation fora)
Solution development	Develop novel policy ideas and solutions by employing methods that foster creativity such as design thinking (policy labs)	Increasing legitimacy by involving citizens directly and by considering the (long-term) implications of new solutions from their perspective (deliberation fora)	Learn from success and failure in view of improving policy implementation in the longer term (policy pilots)

Deliberation fora address the ideas dilemma by integrating the perspectives of citizens and systematically engaging science in deliberation processes. This can facilitate the reassessment of problem definitions and, through the process of knowledge-informed deliberation, develop innovative solutions to complex problems. Pilots contribute to the ideas dilemma insofar as they test policy ideas and iron out teething problems, but are less likely to produce genuinely novel ideas. However, they are aimed at producing ideas to help with policy implementation and solve practical problems. Therefore, as the example of the Innovation Fund illustrates, pilots can be used as a stimulus and vehicle for policy innovation, provided there is a viable mechanism to ensure that learning from piloting is taken forward. In contrast, policy labs are specifically aimed at the ideas dilemma, as they aim to facilitate novel policy solutions shielded from the usual institutional constraints and complexities. By using design thinking or other creative techniques, many policy labs promise to break out of the narrow confines of previous policy approaches and invite fresh thinking to produce creative solutions (Lewis et al. 2019).

Boundary spanning approaches also address the legitimacy dilemma of wicked problems. Policy deliberation fora address the legitimacy dilemma by offering an alternative to conventional political processes. Random selection of participants and inputs from scientific research to inform deliberation enable multiple perspectives to be voiced in complex controversies over wicked problems (*input-legitimacy*) (Farrell et al. 2013; Hammond 2020). It is hoped that successful deliberation processes help make political decision-making better informed (*throughput-legitimacy*) and ultimately improve the quality of decisions (*output-legitimacy*) (Garard et al. 2018; Niemeyer 2014). Policy pilots can also contribute to *throughput-legitimacy* and *output-legitimacy* if they are able to demonstrate a policy’s effectiveness before the policy is rolled out (Ettelt et al. 2015). Preventing ineffective policy from being turned into national policy should also be seen as a success, although politically negative outcomes are more difficult to stomach. Yet, there have also been concerns that piloting depoliticises potentially contestable policy choices by allowing policymakers to claim that implementation is temporary while its effects are meant to last (Ettelt and Mays 2019); however, this was less relevant in our example of the Innovation Fund. Finally, many policy labs aim to increase the legitimacy of policy ideas created in the process by promoting inclusivity, although participation is likely to be selective and, as for the other approaches, there is no guarantee that power imbalances that are inherent in policy processes can be overcome.

Addressing the implementation dilemma is another objective of these boundary spanning approaches. The rationale of policy deliberation fora is to improve the acceptance of policy in society by facilitating transparent and inclusive solution development and by doing so reduce resistance to implementation. Improving implementation is a principal aim of policy piloting. Piloting allows policymakers to manage the risks of policy implementation by testing whether assumptions made in theory hold in practice. It is expected that those participating in pilots will “learn by doing” so that implementation improves incrementally over time. Such gains in knowledge can be multi-dimensional and include relational, cognitive and normative learning (McFadgen and Huitema 2017). In contrast, policy labs do not specifically focus on implementation, although internationally some labs engage in ‘prototyping’ solutions (Buchanan 2018).

Our findings have a number of implications that require further discussion. While we have drawn on current examples of policymaking in Germany, our analysis at this point remains relatively optimistic, given that few examples have shown any definitive results, and our assessment rests on the favourable assumptions reflected in the materials we reviewed. Future research on the subject could also be strengthened by additional field research including interviewing stakeholders and/or participant observation. However, some implications are obvious. All three approaches address complex problems by reducing them to a manageable scale, by ‘localising’ them (townhall meetings), reducing their scope (pilots) or reducing the problem analytically (policy labs). This means that the effects, if there are any, will be small scale, local and project dependent first and foremost. Given the project nature of the approaches these effects may also be temporary only. In the case of fora and labs they may not last beyond the meeting. ‘Rolling out’ in any case is a different ballgame that makes substantially higher demands on skills and resources and brings back the complexity that projects have tried to manage by reduction. Policy labs claim to do this differently by offering to develop creative solutions to problems that can be easily transferred. This may be overly optimistic, as in all likelihood the reductionist approach involved in design thinking and other such techniques is more suitable to solve some problems (e.g. developing a digital application to access a public service) than others (e.g. convince people to change their lifestyles to reduce energy consumption).

All three approaches require careful planning and their outcomes are heavily dependent on how they are executed. Deliberation fora, in particular, are vulnerable to all sorts of dynamics and require attentive facilitation. Likewise, if they are over-engineered, they open themselves to suspicions of ‘window dressing’ and of reproducing biases that they ostensibly seek to overcome. If pilots are badly organised, insufficiently supported and shoddily evaluated, they risk wasting resources rather than contributing relevant policy knowledge. Policy labs using design thinking have been criticised to privilege the designer over other participants and the designer may not be an uninterested party (Iskander 2018).

Another implication is that none of these approaches can ensure that outcomes are transferable or have long-term effects. Clarke and Craft (2019) note that design thinking approaches often underestimate the influence of political and organisational context on the fate of emerging policy ideas. Specifically, their autonomous organisational form, required to ‘unlock creativity’, has the disadvantage that they are often divorced from the political institutions that they seek to influence, and thus lack the capabilities and authority to influence implementation and scale up (Tõnurist et al. 2017; Peters 2020). This raises the question of ownership of policy labs, which is in tension with their proclaimed autonomy. Deliberation fora can only be credible if they produce visible effects that can be experienced by participants and citizens more widely. It will not be enough to publish a final report on a project website to convince future participants to invest their time in politics, local or otherwise. Policy pilots are usually initiated by governments or public sector organisations, as in the case of the Innovation Fund. This should ensure interest and capacity to engage with the findings, yet the approach also requires a viable concept for decision-making post-pilot and transferability. Pilots, if done well, are the most likely candidates to have long-term effects on policy decisions above the local level, but they are also the most expensive tool in terms of time and finance, and they require a long-term strategy to ensure that the gains in expertise and practices are not lost to future users.

This paper contributes to the literature on boundary spanning activities by expanding the concept to current approaches to improving policy processes. These findings are also relevant to inform current debates about the direction of policymaking on wicked problems and are aimed to help inform choices, and moderate expectations. Based on our findings, we have concluded that each approach uses boundary spanning activities to promote its aims while reflecting their different priorities. They can be helpful to address wicked problems, including by involving science and research and making scientific expertise relevant at different stages of the policy process. However, there is no guarantee that they will have much effect beyond the duration of the project and each of them requires resources, careful planning and execution, and a supportive organisational environment to fulfil its potential in contributing to policy.

## Supplementary Information

Below is the link to the electronic supplementary material.

Supplementary file1 (DOCX 48 kb)
