# Supplementary material for: Spanning the boundaries between policy, politics and science to solve wicked problems: policy pilots, deliberation fora and policy labs
Source: Sustain Sci. 2022 Aug 23;18(2):809–21. doi: 10.1007/s11625-022-01187-y (PMC9395888; doi:10.1007/s11625-022-01187-y)
Supplement: Supplementary file 1 — Supplementary file1 (DOCX 48 kb) [file 11625_2022_1187_MOESM1_ESM.docx]

**Supplementary Materials**

**Appendix 1: Flow diagram for the case selection**

**Identification of studies via Scopus and Factiva databases**

Records removed *before screening*:

Records marked as ineligible by automation tools (duplicate records, NOT Germany, before 2019, companies, other languages than EN and DE)

Records identified from*:

**Scopus Database**

- Policy Pilot* (n=103)
- Policy Lab**(n=42)
- Deliberation***

**Factiva Database**

- Policy Pilot* (n=754)
- Policy Lab** (n=63)
- Deliberation*** (n= 965)

**Identification**

Records excluded because the did not entail relevant information

**Scopus Database**

- Policy Pilot* (n=13)
- Policy Lab** (n=10)
- Deliberation*** (n=145)

**Factiva Database**

- Policy Pilot* (n= 288)
- Policy Lab**(n = 41)
- Deliberation*** (n=307)

Records screened and sought for retrieval

**Scopus Database**

- Policy Pilot* (n=26)
- Policy Lab** (n=13)
- Deliberation*** (n=149)

**Factiva Database**

- Policy Pilot* (n=433)
- Policy Lab** (n=54)
- Deliberation*** (n=461)

**Screening**

Reports excluded: (no information on the functioning of instruments/ no link to science)

Reports assessed for eligibility

**Scopus Database**

- Policy Pilot* (n=13)
- Policy Lab** (n=3)
- Deliberation*** (n=4)

**Factiva Database**

- Policy Pilot* (n=145)
- Policy Lab** (n=13)
- Deliberation*** (n=154)

Studies included in review

**Scopus Database**

- Policy Pilot* (n=4)
- Policy Lab** (n=2)
- Deliberation*** (n=2)

**Factiva Database**

- Policy Pilot* (n=65)
- Policy Lab** (n=11)
- Deliberation*** (n=129)

**Included**

***Policy pilots:** Factiva: policy pilot or Reallabor or Pilotstudie and (health or climate or Gesundheit or Klima) and (forsch* or expert* or wissenschaft*) // Scopus: ( germany AND policy AND pilots AND health OR climate )

****Policy labs**: Factiva: policy lab or policy design or lab and (health or climate or Gesundheit or Klima) and (forsch* or expert* or wissenschaft*) and Germany // Scopus: policy lab and (health or climate or Gesundheit or Klima) and (forsch* or expert* or wissenschaft*)

*****Policy deliberation forum**: policy deliberation or deliberation or Bürgerforum or townhall or mini public and (health or climate or Gesundheit or Klima) and (forsch* or expert* or wissenschaft*) and Germany // Scopus: policy deliberation or or Bürgerforum or townhall or mini public and (health or climate or Gesundheit or Klima) and (forsch* or expert* or wissenschaft*) and Germany

*Inspired from:*  Page MJ, McKenzie JE, Bossuyt PM, Boutron I, Hoffmann TC, Mulrow CD, et al. The PRISMA 2020 statement: an updated guideline for reporting systematic reviews. BMJ 2021;372:n71. doi: 10.1136/bmj.n71

**Appendix 2: Boundary spanning activities identified with the search of Scopus and Factiva databases**

* Type of boundary spanning activity: Policy Lab (PL); Policy Pilot (PP); Deliberation Forum (DF) as defined in the paper

** Policy field: H (health policy); E (environment/climate policy)

***The date refers to the time when the boundary spanning activity took place, as described in public documents. The list does not include boundary spanning activities that are only announced but that have not started as of November 2021.

**** Level of implementation (national, regional local), i.e. level for which the boundary spanning activity is conceptualized and at which it is realized. Boundary spanning activities at the national level generally include local events across the country that are linked as parts of a national framework for boundary spanning. They are dealing with health or climate issues that concern not only a community of a region but national politics. Similarly, regional activities generally take place at a certain local place but they have a regional scope, generally tackling health or climate problems at the level of the federal states (Laender). Local boundary spanning activities are oriented towards

***** The issue focus summarizes the main health or climate policy issue that is addressed with the boundary spanning activity

******* The link to science differs across the boundary spanning instruments. It is considered to be strong if scientists are systematically included in the process. The link is considered to be average, if there is some (but unsystematic) expert support in the process. The link is considered to be weak if scientists are only project partners or those who have contributed to the conceptualisation/evaluation of the project without taking part in the activities as such.

|  | Name | Type of instrument* | Policy field ** | Date *** | Level of implementation**** | Issue focus ***** | Link to Science****** |
| --- | --- | --- | --- | --- | --- | --- | --- |
| 1 | **Bürgerrat Klima** | DF | E | 2020-2021 | National level | Climate Governance Germany | Strong, Science Council, including 25 scientists, evaluation |
| 2 | **Bürgerdialog Klimaschutz** | DF | E | 2015 | Regional level | Climate Protection Germany, Paris Agreement | Strong, scientific advisory board assessed the results |
| 3 | **Bürgerforen Bonn** | DF | E | 2020 - 2022 | Local level | Climate Governance, Ecological Transition | Strong, process support, evaluation, expert participants |
| 4 | **Demokratiekonvent FFM** | DF | E | 2019 and 2021 | Local level | Climate politics (2021); Public participation (2019) | Strong, University Frankfurt; Evangelische Academy Frankfurt |
| 5 | **Bürgerrat Klima Offenburg** | DF | E | 2021 | Local level | Climate Politics: Mobility, Energy, Sustainable Lifestyle | Strong, experts support meetings with knowledge on topics |
| 6 | **Bürgerräte BioökonomiereREVIER** | DF | E | 2021 | Regional level | Bioeconomy | Strong; Ruhr-University Bochum provides input and evaluation |
| 7 | **Bürgerrat Klima Augsburg** | DF | E | 2021 | Local level | Climate Protection | Strong, University Augsburg input and evaluation |
| 8 | **Bürgerforum Corona Baden-Württemberg** | DF | H | 2020-2021 | Regional level | Care, Corona, testing, vaccination, post-covid | Strong, expert input, evaluation, participating experts |
| 9 | **Thüringer Bürger Forum** | DF | H | 2021 | Regional level | Corona pandemic, Health crises, health policy | Average, Experts are invited and provide input upon request |
| 10 | **Bürgerbeirat Gesundheitsregion PLUS Landkreis Dachau** | DF | H | 2021 - 2022 | Regional level | Health policy | Average, Experts are invited and provide input upon request |
| 11 | **Bürgerforum „Alters-vorsorge der Abgeordneten“** | DF | H | 2017-2018 | Regional level | Health policy, Retirement | Average, Experts are invited and provide input upon request |
| 12 | **Bergisch Gladbach: Bürgerrat Klima** | DF | E | 2021 - 2022 | Local level | Climate change | Average, Experts are invited and provide input upon request |
| 13 | **Corona Forum / Bürgerrat Sachsen** | DF | H | 2021 | Regional level | Covid Pandemic, health policy | Average, Experts are invited and provide input upon request |
| 14 | **Bürgerrat "Nachhaltiges Leben in Jülich"** | DF | E | 2021 | Local level | Climate change, environment | Strong, process support, evaluation, expert participants |
| 15 | **Temporäre Radfahrstreifen während Corona-Krise** | PP | E | 2020 | Local level | Mobility | Weak, Concept and evaluation |
| 16 | **Kommunale Klimaschutz-Modellprojekte im Rahmen der Nationalen Klimaschutzinitiative** | PP | E | 2018/2020 | Local level | Climate mitigation | Weak, Possible project partner, evaluation |
| 17 | **Modellprojekt Demenzatlas Hessen** | PP | H | 2016-2021 | Local level | Health, aging | Average, expert input and evaluation |
| 18 | **Pilotprojekt Berliner Philharmoniker Einlass nur nach negative Corona-Test** | PP | H | 2021 | Local level | Culture, testing, corona | Weak, Concept and evaluation |
| 19 | **Bayerische Staatsoper** | PP | H | 2020 | Local level | Culture, testing, corona | **Strong,** Technische Universität München / Landesamt für Gesundheit und Lebensmittelsicherheit |
| 20 | **Modellprojekte zur Stärkung des öffentlichen Personennahverkehrs** | PP | E | 2021 | Local level | Mobility | Weak, Possible project partner |
| 21 | **Sachsen Pilotprojekt Corona-Impfungen** | PP | H | 2021 | Local level | Vaccination | Weak, Concept design |
| 22 | **Corona Pilotprojekt Tübingen** | PP | H | 2021 | Local level | Opening, post Covid | Weak, Concept design |
| 23 | **Innovationsfonds** | PP | H | 2016-2019 | National level | Health care | Strong, expert input and scientific evaluation of pilot projects |
| 24 | **Pilotprojekte für die Post-Corona-Stadt** | PP | H | 2020-2023 | Local level in Aachen, Erlangen, Weimar, Ludwigsburg, Mannheim, München, Neuruppin, Berlin, Frankfurt am Main | post Covid politics | Average, Experts are invited and provide input upon request (depends on sub-project) |
| 25 | **Media Policy Lab** | PL |  | 2017-ongoing | Local level | Digitalisation | Strong, process support, evaluation, expert participants |
| 26 | **European Policy Labs** | PL | E/H | 2019 - 20 | Local level | Inclusive Growth in Europe | Average, expert input and evaluation |
| 27 | **The Policy Lab** | PL | E/H | Since 2013 | Local level | Political participation | Strong, Leuphana University |
| 28 | **Social2Mobility** | PL | E |  | Local level | Mobility | Strong, process support, evaluation, expert participants |
| 29 | **Social Lab Nutztiere** | PL | E | 2015-2019 | Local level | Food, Animal rights | Strong, process support, evaluation, expert participants |
| 30 | **Sustainable Accessible Innovations Laboratory (SAI-Lab)** | PL | E | 2017-ongoing | Local level | Sustainability Innovation | Strong, TU Berlin |
| 31 | **Quartier Zukunft – Labor Stadt** | PL | E | 2011-2020 | Local level | Climate change, Resources, City | Strong, KIT / EIFER |
| 32 | **INTERPART** | PL | E | 2018-2021 | Local level | Cities, participation | Strong, 2 universities |
| 33 | **i Resilience** | PL | E | 2019-ongoing | Local level | Climate change | Strong, process support, evaluation, |
| 34 | **Reallabore Energiewende** | PL | E | 2019-ongoing | Regional level (8 sub-labs in different regions) | Energy transition | Weak, evaluation since 2021 |
| 35 | **Innovation Lab zu Mobilitätswende** | PL | E | 2016 | Local level | Sustainable Energy | Average, expert input and evaluation |
| 36 | **Biodiversity Policy Lab** | PL | E | 2021 | National level | Biodiversity | Strong, process support, evaluation, expert participants |
| 37 | **Digitalisierungslabore** | PL | E/H | 2017-2022 | National level | Digitalisation | Strong, process support, evaluation, expert participants |
| 38 | **Klimaanpassung Peer-Learning-Werkstätten** | PL | E | 2019-2022 | Local level | Climate adaptation | Average, expert input and evaluation |
